# Distribución de la fuerza de trabajo en enfermería en la Región de las Américas

**DOI:** 10.26633/RPSP.2019.319

**Published:** 2019-09-25

La *Revista Panamericana de Salud Pública* llama la atención a los lectores sobre un error en el siguiente artículo, señalado por los autores:

Cassiani SHB, Hoyos MC, Barreto MFC, Sives K, da Silva FAM. Distribución de la fuerza de trabajo en enfermería en la Región de las Américas. Rev Panam Salud Publica. 2018;42:e72. https://doi.org/10.26633/RPSP.2018.72

En la página 3, cuadro 1, el número de enfermeros por 10 000 habitantes en Argentina (2015) es 42,4 en lugar de 4,24.

**Table 1:** 

Cono Sur	14,80	0,60	81 555	17,3	216 877	46,1	171 911	36,6	470 343
Argentina (2015)	42,4	0,56	19 729	11,0	73 373	41,0	86 073	48,0	179 175

